# Therapeutic alliance and symptom change in enhanced cognitive-behavioural therapy for eating disorders

**DOI:** 10.1186/2050-2974-1-S1-O9

**Published:** 2013-11-14

**Authors:** Bronwyn Raykos, Peter McEvoy, Anthea Fursland, Sharon Ridley, Paula Nathan

**Affiliations:** 1 Centre for Clinical Interventions, Australia

## Objective

To investigate the role of therapeutic alliance in cognitive-behavioural therapy enhanced (CBT-E) for eating disorders.

## Method

200 individuals (16+ years) admitted to the CBT-E treatment program at the Centre for Clinical Interventions completed the Helping Alliance Questionnaire-2nd Edition (HAQ-II; Luborsky et al., 1996) and the Eating Disorder Examination-Questionnaire (EDE-Q; Fairburn & cooper, 1993) at the start, middle, and end of treatment as well as baseline and post-treatment measures of related pathology.

## Results

Scores on the HAQ-II increased significantly from the start to the middle of treatment and were maintained post-treatment. Alliance was not related to improvement in eating disorder symptoms at any time point or to drop-out. For patients who completed CBT-E, lower scores on the EDE-Q at post-treatment were associated with more positive ratings of alliance at the end of treatment.

## Discussion

CBT-E, a manual-based treatment for eating disorders, yields excellent patient ratings of alliance at various time points. Overall, alliance was unrelated to eating disorder pathology or to changes in symptoms although a greater reduction in symptoms at the end of treatment was associated with more positive ratings of alliance at the end of treatment. There was no evidence that alliance drives symptom change in CBT-E.

This abstract was presented in the **Adult Treatment and Services** stream of the 2013 ANZAED Conference.

